# Amplifying Women's Voices in Menopause Research: The Importance of Inclusive Perspectives

**DOI:** 10.1111/hex.70163

**Published:** 2025-01-22

**Authors:** Camille Cronin, Sara Donevant, Kerri‐ann Hughes, Marja Kaunonen, Jette Marcussen, Rhonda Wilson

**Affiliations:** ^1^ School of Health and Social Care University of Essex Colchester Essex UK; ^2^ College of Nursing University of South Carolina Columbia USA; ^3^ School of Nursing Massey University Palmerston North New Zealand; ^4^ School of Health Sciences Tampere University Tampere Finland; ^5^ Department of Applied Health Sciences UCL University College Odense Denmark; ^6^ Department of Nursing RMIT University Melbourne Australia

**Keywords:** experience, health disparities, inclusive research, menopause, participatory research, women's voices

## Abstract

Menopause, a significant life transition for half the global population, intersects biological, cultural and social dimensions. Despite its universal occurrence, menopause research has historically been dominated by biomedical perspectives, often neglecting women's voices and diverse experiences. This article highlights the importance of including women's perspectives in menopause research to ensure relevance, accuracy and equity. It explores cultural variations in menopause experiences, the impact of socioeconomic status and the often‐overlooked emotional and psychological dimensions. This article advocates for participatory approaches, emphasising that women's involvement enhances research design, implementation and policy development. Evidence‐based interventions that are tailored to the individual can better address the diverse needs of menopausal women. The discussion extends to addressing health disparities, calling for inclusive research and policies to ensure equitable access to care. Ultimately, empowering women through co‐designed research and inclusive policies can transform the menopause journey into an enriching life phase, promoting resilience and inclusivity across communities. This paradigm shift in menopause research and policy highlights the need for diversity, collaboration and evidence‐based approaches to improve health outcomes and quality of life for all women.

**Patient or Public Contribution:**

This article has been informed by a menopause service user group who discuss their experiences of menopause. The group was formed because of initial qualitative research and now meet on a regular basis to co‐design and co‐produce activities that inform ongoing research for the menopause taskforce.

Menopause is very much in the media with discourse on TV, in the press and in social media. Menopause represents a significant transitional period in the lives of women, intersecting biological changes with societal and personal dimensions [1]. When the discomfort of menopause impacts everyday life, either at home or work, then this needs attention [2]. Research on menopause has frequently been approached through a predominantly biomedical approach, overshadowing the voices, autonomy and diverse experiences of women. In this article, we present five reasons why the voices of women should be heard in women's health and particularly menopause research.

## 1 The Crucial Role of Women's Voices in Menopause Research

Menopause is an inevitable life stage for half the world's population, but experiences vary hugely [3]. It is often accompanied by a complex array of symptoms and changes that can affect physical, emotional and social well‐being. Menopause marks the end of a woman's reproductive stage when the ovaries stop functioning well, hormone levels fall and periods cease. Menopause is diagnosed when there have been no periods for 12 months. The menopause typically happens between the ages of 45 and 55, although this varies slightly by geographic region. For example, in the UK, the average age of the menopause is 51. However, this may vary globally due to differences in factors such as diet, lifestyle, culture and health. For instance, women in Southeast Asia report fewer vasomotor symptoms but greater musculoskeletal complaints compared to their Western counterparts. Black and Asian women tend to experience more intense and frequent hot flushes than white women, yet there is limited understanding of why this is the case. Similarly, Latina women often view menopause as a positive transition into a new life stage, but these cultural attitudes towards menopause vary, influencing how symptoms are perceived and addressed and are often overlooked in mainstream research.

Despite its universal occurrence, menopause has historically been under‐researched and inadequately addressed within healthcare systems. However, the tide is beginning to change as the importance of incorporating women's voices in menopause research becomes increasingly recognised. Women's perspectives are vital not only for enhancing the relevance and accuracy of research but also for driving the development of evidence‐based policies and healthcare services that can genuinely meet their needs [3]. Tailored interventions that respect cultural diversity are essential for equitable care [2].

## 2 Capturing the Full Spectrum of Experiences in Menopause

Every woman's experience with menopause is unique, influenced by factors such as genetics, lifestyle, culture and overall health [3]. While some women may pass through menopause with minimal discomfort, others may experience severe symptoms such as hot flushes, night sweats, mood swings, sleep disturbances and cognitive changes. These symptoms can significantly impact a woman's quality of life, yet they are often underreported and poorly understood.

Women's voices are essential in capturing the full spectrum of these experiences in research [2]. By directly involving women in menopause research, whether through surveys, focus groups or participatory studies, researchers can gain a deeper understanding of the varied and nuanced ways menopause affects different individuals. This input is invaluable for ensuring that the research reflects real‐world scenarios and addresses the actual concerns of women going through menopause [4]. Without this input, research risks being incomplete or biased, potentially leading to ineffective or irrelevant conclusions that misinform practice. So actively involving women in the research process means working with the researchers from the formulation of research questions to the design and implementation of studies, which leads to a more meaningful approach [2]. Women are best positioned to identify the issues that matter most to them, such as the impact of menopause on their work lives, relationships and mental health. This participatory approach ensures that the research studies conducted are not only scientifically rigorous but also directly aligned with the needs and priorities of those who will benefit from the research [3].

Moreover, women's involvement can lead to the discovery of previously overlooked aspects of menopause. For instance, while the physical symptoms of menopause are often highlighted, the emotional and psychological effects, such as anxiety, depression and a sense of loss, may not receive the same attention [5]. Women's voices can help bring these issues to the forefront, encouraging a more holistic approach to menopause research that considers all dimensions of a woman's health.

## 3 Developing Effective Treatments and Interventions for Menopause

The insights gained from women's participation in menopause research are crucial for the development of effective treatments and interventions. Understanding women's preferences, concerns and expectations can guide the creation of therapies that are not only effective but also acceptable and accessible to the women who need them [1]. For example, some women may prefer non‐hormonal treatments due to concerns about the risks associated with menopausal hormone therapy (MHT), also known as hormone replacement therapy (HRT), while others may prioritise treatments that address specific symptoms such as sleep disturbances or mood swings [2].

By incorporating women's feedback into clinical trials and treatment development, healthcare providers can offer a broader range of options that cater to the diverse needs of women experiencing menopause. Other research approaches incorporating a participatory approach and qualitative approaches promote a more person‐centred approach in menopause which increases the likelihood that women will adhere to treatments either through pharma or non‐pharmacological interventions and experience positive outcomes [1], addressing the discomforts of menopause and ultimately improving their quality of life during this transitional period [5]. The recent updated clinical guidance from the National Institute for Health and Care Excellence updated the guidance on menopause management and recommends cognitive behaviour therapy (CBT) as a proven intervention for depression and anxiety across life stages and is effective for sleep disturbance and for vasomotor symptoms. CBT is specifically recommended for depressed mood during menopause. The North American Menopause Society position statement also recommended CBT for bothersome vasomotor symptoms.

## 4 Informed Decision‐Making in Women's Health

Evidence‐based policymaking is a critical component of effective healthcare, particularly in areas like women's health, where historically, there has been a lack of focus and investment. For menopause, evidence‐based policies are essential for ensuring that healthcare services and resources are directed towards the most pressing needs and that interventions are grounded in scientific evidence rather than anecdotal or outdated information.

When policies are based on robust evidence, decision‐makers are equipped with the information they need to make informed choices that benefit women's health. This is particularly important in menopause care, where misconceptions and stigma can sometimes influence policy and practice [4]. By relying on data from well‐conducted research, policymakers can develop guidelines and programs that address the real challenges women face during menopause rather than perpetuating myths or ignoring the issue altogether.

For instance, evidence‐based policies such as the Women Health Strategy implemented in England have led to the localised review of services and using community voices to shape women's health services to ensure that services are planned with women and for women, so they have access to the support they need. While this is very much developing, the idea of the end user being involved through the evaluation will add benefit. Additionally, such policies have led and promoted the increased availability of training of healthcare professionals in menopause management, and improving the quality of care that women receive. In 2024, in England, the Health and Social Care Secretary named problem periods, women's health research and support for domestic and sexual abuse victims among the government's priorities for women's health.

## 5 Addressing Health Disparities in Women's Health

Evidence‐based policymaking also plays a crucial role in addressing health disparities and ensuring that all women, regardless of their socioeconomic status, ethnicity or geographic location, have access to appropriate menopause care. Research has shown that women from marginalised communities often experience greater barriers to accessing healthcare, including menopause‐related services [2]. By focusing on evidence, policymakers can identify these disparities and implement targeted interventions to bridge the gap. For example, policies could support community‐based programs that provide education and resources to underserved populations, or fund research that explores the specific needs of minority groups during menopause. Such initiatives and community voices would help ensure that menopause care is inclusive and equitable, reaching all women who need it.

In other research, socioeconomic status has been shown to influence menopause experience and the workplace, which makes a compelling case for the need to address systemic inequities through targeted workplace policies and research initiatives [4]. There is still much work to do in this area. As such research is limited, where there is a heavy reliance on existing research focused predominantly on higher socioeconomic status women, which constrains the ability to draw robust conclusions about the lower socioeconomic status across diverse populations. This is why a critical lens is required on how socioeconomic disparities exacerbate the challenges menopausal women face in the workplace. It highlights the urgent need for inclusive research, recognition of workplace accommodations and education to reduce inequities.

## 6 Conclusion

Incorporating women's voices in the design and roll out of menopause research and ensuring that interventions, policies and services are evidence‐based are essential steps toward improving the health and well‐being of women during menopause. Empowering women through menopause research and policy is a multidimensional endeavour, requiring collaboration across healthcare, workplace and societal domains. By adopting a co‐design and co‐production approach to participatory research, stakeholders can redefine menopause as a transformative and enriching phase of life. This approach not only benefits individual women but also strengthens communities and economies by fostering resilience and inclusivity. Future efforts must prioritise diversity, inclusivity and the active involvement of women in shaping their menopause experiences.

By prioritising the experiences and needs of women, research, policies and health and social services can be developed that truly support women through this important life stage, leading to better health outcomes and enhanced quality of life.

## Author Contributions

**Camille Cronin:** conceptualization, writing–original draft, writing–review and editing, project administration. **Sara Donevant:** writing–review and editing. **Kerri‐ann Hughes:** writing–review and editing. **Marja Kaunonen:** writing–review and editing. **Jette Marcussen:** writing–review and editing. **Rhonda Wilson:** writing–review and editing, conceptualization.

## Ethics Statement

The authors have nothing to report.

## Consent

The authors have nothing to report.

## Conflicts of Interest

The authors declare no conflicts of interest.

## Data Availability

The authors have nothing to report.
